# Supplementary material for: Mycobacterium dormancy and antibiotic tolerance within the retinal pigment epithelium of ocular tuberculosis
Source: Microbiol Spectr. 2024 Jun 25;12(8):e00788-24. doi: 10.1128/spectrum.00788-24 (PMC11302011; doi:10.1128/spectrum.00788-24)
Supplement: Supplemental material — Fig. S1 to S7. [file spectrum.00788-24-s0001.pdf]

Supplementary Information (SI) for

Mycobacterium dormancy and antibiotic tolerance within the retinal pigment epithelium of ocular tuberculosis

Rachel Liu[1], Joshua N. Dang[2], Rhoeun Lee[1,3], Jae Jin Lee[1], Niranjana Kesavamoorthy[2], Hossein Ameri[2], Narsing Rao[2], Hyungjin Eoh[1,2,*]

[1]Molecular Microbiology and Immunology, Keck School of Medicine, University of Southern California, Los Angeles, CA

[2]Roski Eye Institute, Keck School of Medicine, University of Southern California, Los Angeles, CA

[3]School of Pharmacy, Sungkyunkwan University, Suwon, Republic of Korea

*, Corresponding author: Hyungjin Eoh, 1501 San Pablo Street, ZNI 537, Los Angeles, California 90033. Phone: 1-323-442-6048. Email: heoh@usc.edu

Supplementary Figures

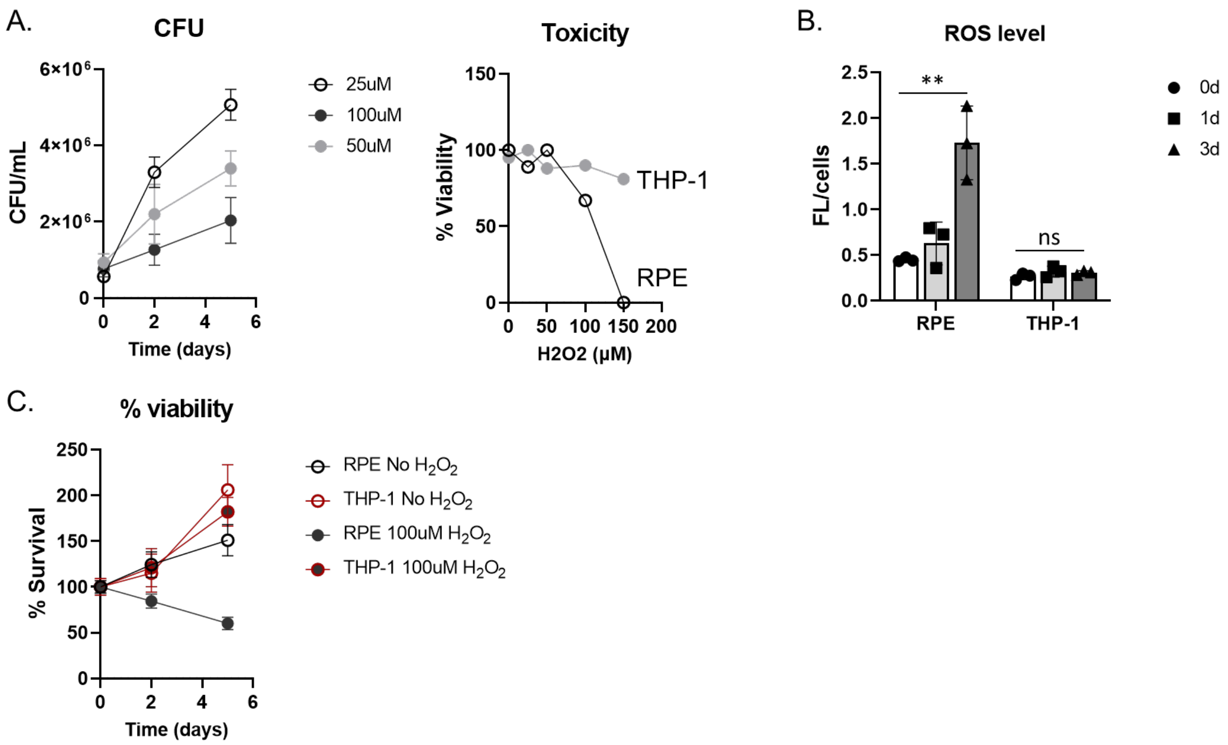

S Fig. 1. Development of *in vitro* RPE cell culture system to model *in vivo* mycobacterial dormancy within the RPE cells. **A**. (Left panel) H37Ra CFU viability following treatment with 25, 50, or 100 μM H_2O_2 *in vitro* culture media (Right panel) Percentage viability of RPE cells and THP-1 macrophages following treatment with various concentrations of H_2O_2 for 24 hours. **B**. ROS accumulation within RPE cells or THP-1 macrophages following treatment with 50 μM H_2O_2 for 0, 1, or 3 days. **C**. % CFU viability of intracellular H37Ra following infection of RPE cells or THP-1 macrophages treated with 100 μM H_2O_2. **, $P<0.01$; ns, not significant by Student t-test.

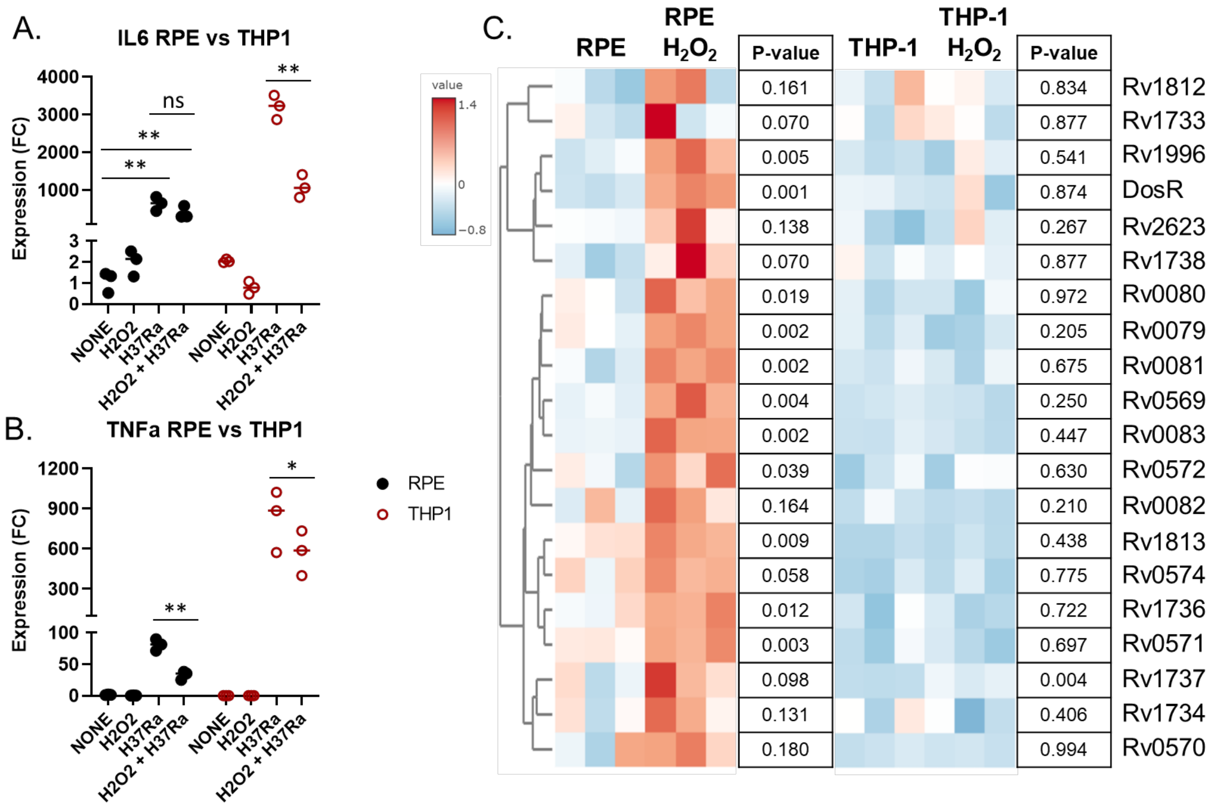

S Fig. 2. RPE cell or THP-1 macrophage mRNA transcript levels of proinflammatory cytokine genes (**A**. *il6* and **B**. *tnfα*) following treatment with 50 μM H_2O_2 and/or infection with H37Ra for 24 hours. FC, fold change. **C**. A total 19 DosR regulon mRNA expression levels in either RPE cells or THP-1 macrophages after treatment with 50 μM H_2O_2 for three days. DosR regulon genes were selected as previously reported. (https://doi.org/10.3389/fbioe.2013.00004). P-values were calculated by comparing the expression levels in H_2O_2 treated condition with those in an untreated condition. *, $P<0.05$; **, $P<0.01$; ns, not significant by Student t-test.

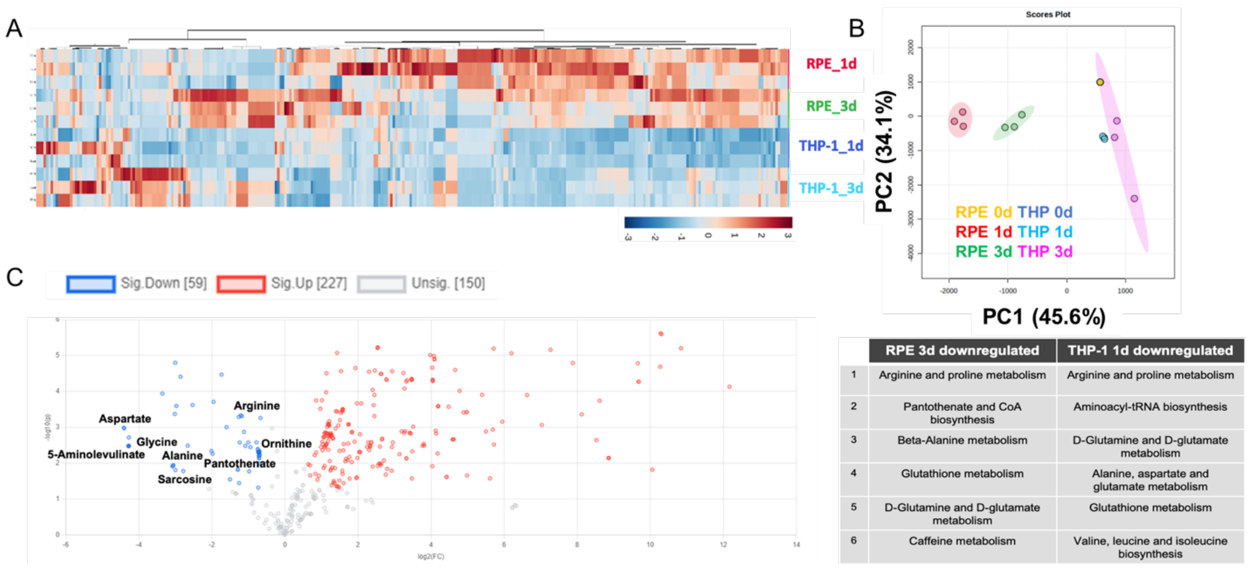

	RPE 3d downregulated	THP-1 1d downregulated
1	Arginine and proline metabolism	Arginine and proline metabolism
2	Pantothenate and CoA biosynthesis	Aminoacyl-tRNA biosynthesis
3	Beta-Alanine metabolism	D-Glutamine and D-glutamate metabolism
4	Glutathione metabolism	Alanine, aspartate and glutamate metabolism
5	D-Glutamine and D-glutamate metabolism	Glutathione metabolism
6	Caffeine metabolism	Valine, leucine and isoleucine biosynthesis

S Fig. 3. **A**. Clustered heat map depicting levels of approximately 430 metabolites of infected RPE cells and THP-1 macrophages treated with 50 µM H_2O_2 at 1 and 3 days post-infection. Rows depict cell type and timepoints and columns indicate individual metabolites. Data are depicted on a log2 scale relative to the 0 day metabolite abundance. **B**. Principal Component Analysis (PCA) of the metabolome profiles of infected RPE cells and THP-1 macrophages with treatment of 50 µM H_2O_2. Metabolites were extracted at 0, 1, and 3 days post-infection. **C**. Volcano plot depicting up- and down-regulated pathways in infected RPE cells treated with 50 µM H_2O_2 for 3 days. Statistically over- and under-represented pathways were screened by log[P] values and pathway impact scores. Table indicates the pathway mapping result. The analysis identified the metabolic pathways altered the most in RPE cells (day 3) or THP-1 macrophages (day 1) post-infection with H37Ra.

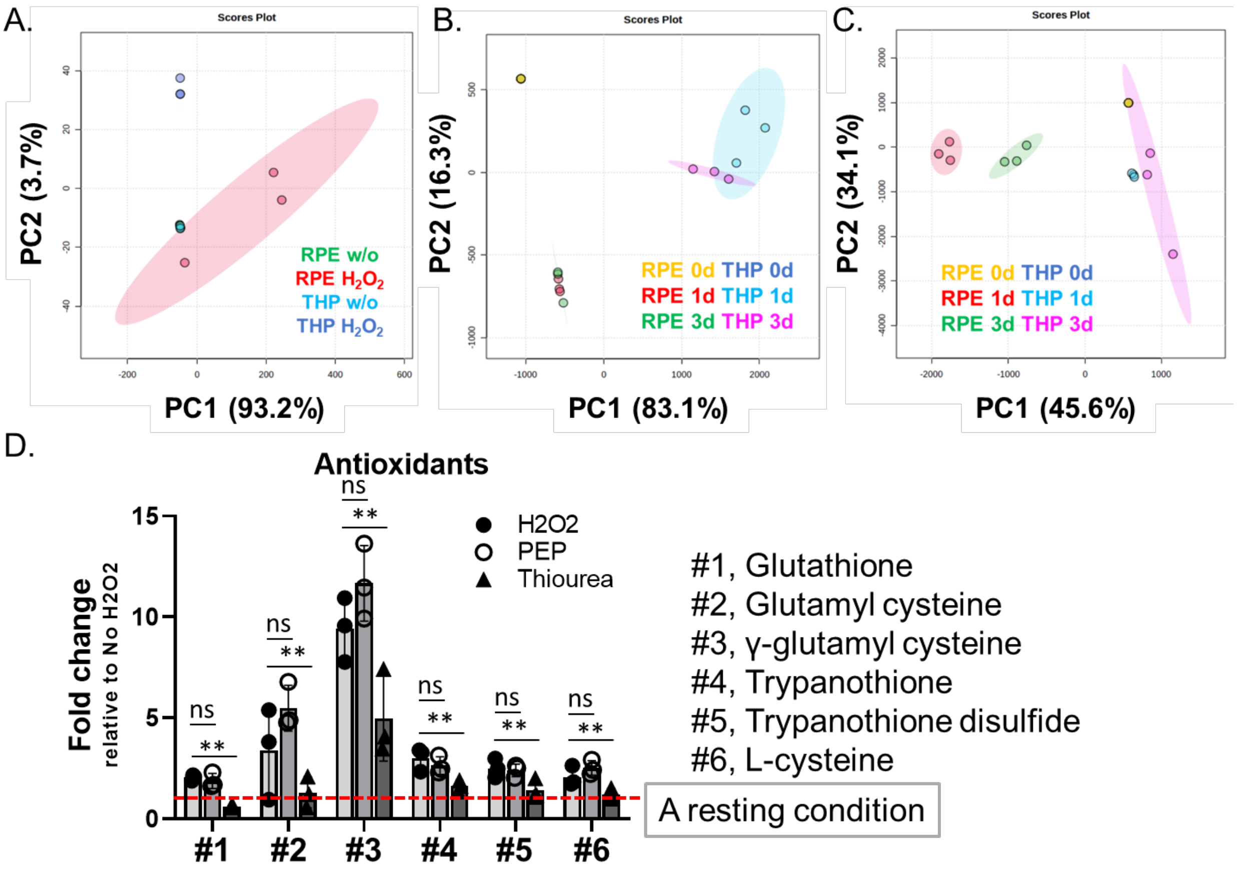

S Fig. 4. Metabolomics analysis of RPE cells and THP-1 macrophages. PCA analysis of metabolome profiles of RPE cells and THP-1 macrophages (**A**) after treatment with 50 μM H_2O_2 only, (**B**) infection with H37Ra only, and (**C**) infection with H37Ra and treatment with 50 μM H_2O_2. Metabolites were extracted at days 0 and 1 post-treatment with H_2O_2 and days 0, 1, and 3 post-infection with H37Ra. **D**. Targeted metabolomics analysis of infected RPE cells treated with 50 μM H_2O_2 (black circles) with additional supplementation with 10 mM PEP (open circles) or 10 mM thiourea (black triangles) have been focused on metabolites involved in the antioxidant pathways. All values are depicted by fold changes relative to that of untreated controls. All values are the average ± SEM; *, $P < 0.01$; ns, not significant by Student's t test.

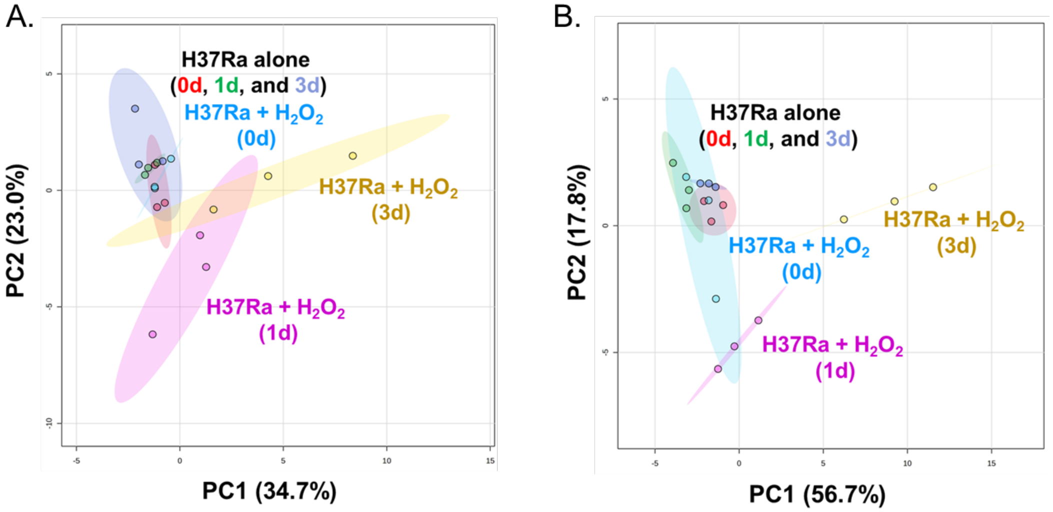

S Fig. 5. Intracellular metabolic state of RPE cells after co-treatment with H_2O_2 and H37Ra infection. A/B Principal component analysis (PCA) of the lipidomics profiles of infected THP-1 macrophages (A) and RPE cells (B) with or without treatment with 50 μM H_2O_2. Metabolites were collected at days 0, 1, and 3 post-infection. Red and Blue spheres, triplicate samples of cells before infection or treatment with H_2O_2. Green and light purple spheres, infected cells at days 1 and 3 post-infection, respectively. Pink and yellow spheres, infected cells treated with H_2O_2 at days 1 and 3 post-infection, respectively.

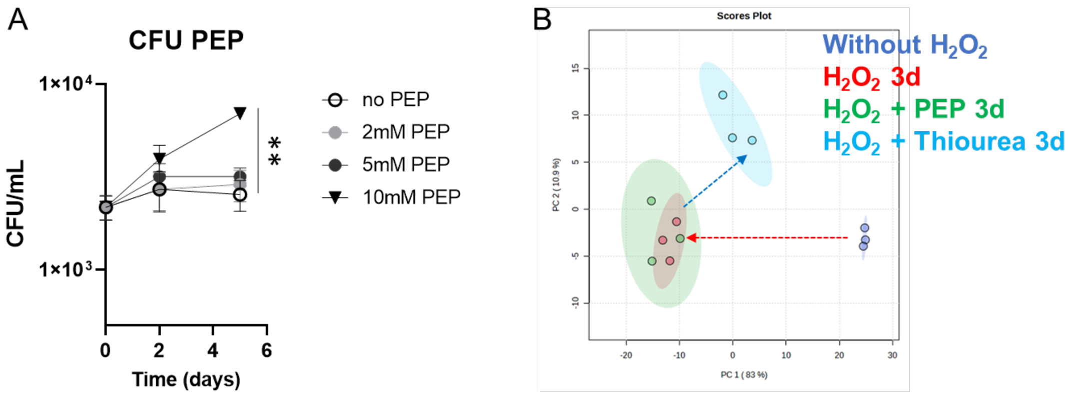

S Fig. 6. The effect of PEP treatment on intracellular *M. tuberculosis* and RPE cells. **A.** CFU viability of H37Ra with supplementation of 0 – 10 mM PEP. **B.** PCA of the metabolome profiles of infected RPE cells treated with 50 μM H_2O_2 (red spheres) with additional supplementation of 10 mM PEP (green spheres) or 10 mM thiourea (light blue spheres). Metabolites were extracted at 0 (dark blue) and 3 days post-infection. PEP treatment showed little impact on metabolic networks of H_2O_2 treated RPE cells, while thiourea treatment changed the metabolism towards that without treatment. **, P<0.01 by Student t-test.

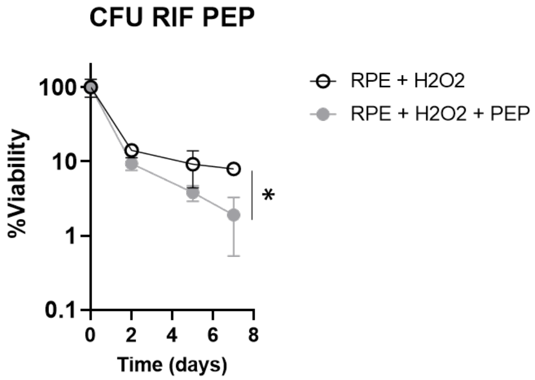

S Fig. 7. % viability of intracellular HN878 within RPE cells. CFU viability of intracellular HN878 within RPE cells following co-treatment with 50 μM H_2O_2 and 5x MIC-equivalent RIF was used to calculate % viability relative to the number of initial viable bacilli. *, P<0.01 by ANOVA.
